# Diabetes and Obesity—Cumulative or Complementary Effects On Adipokines, Inflammation, and Insulin Resistance

**DOI:** 10.3390/jcm9092767

**Published:** 2020-08-26

**Authors:** Adela-Viviana Sitar-Taut, Sorina Cezara Coste, Simina Tarmure, Olga Hilda Orasan, Adriana Fodor, Vasile Negrean, Dana Pop, Dumitru Zdrenghea, Cezar Login, Brandusa Tiperciuc, Angela Cozma

**Affiliations:** 1 Internal Medicine Department, 4th Medical Clinic “Iuliu Haţieganu” University of Medicine and Pharmacy, 400012 Cluj-Napoca, CJ, Romania; secara.sorina@yahoo.com (S.C.C.); siminatarmure@yahoo.com (S.T.); olgaorasan@yahoo.com (O.H.O.); vasile.negrean@umfcluj.ro (V.N.); angelacozma@yahoo.com (A.C.); 2 Clinical Center of Diabetes, Nutrition, Metabolic diseases, “Iuliu Haţieganu” University of Medicine and Pharmacy, 400012 Cluj-Napoca, CJ, Romania; adifodor@yahoo.com; 3 Department of Cardiology, Clinical Rehabilitation Hospital, “Iuliu Haţieganu” University of Medicine and Pharmacy, 400012 Cluj-Napoca, CJ, Romania; pop67dana@gmail.com (D.P.); dzdrenghea@yahoo.com (D.Z.); 4 Department Physiology, “Iuliu Haţieganu” University of Medicine and Pharmacy, 400012 Cluj-Napoca, CJ, Romania; cezar.login@umfcluj.ro; 5 Department Pharmaceut Chem “Iuliu Haţieganu” University of Medicine and Pharmacy, 400012 Cluj-Napoca, CJ, Romania; brandu32@yahoo.com

**Keywords:** diabetes, obesity, adipokines, inflammation, insulin resistance score

## Abstract

Background: Diabetes and obesity are increasingly significant public health issues. The aim of this study was to evaluate the relationship between adipocytokines (leptin, ghrelin, and chemerin), inflammation (sVCAM1—soluble vascular adhesion molecule 1, sICAM1—soluble intercellular adhesion molecule 1), and insulin resistance in the presence of obesity and diabetes mellitus. Methods: 88 subjects, with a mean age of 61.96 ± 10.15 years, 75% of whom were women, were evaluated (in order to consider different associations between obesity and diabetes, subjects were categorized into four groups). Results: Overall, we found significant correlations between sICAM1-sVCAM1 rho = 0.426 and ghrelin-chemerin rho = −0.224. In the obesity + diabetes group, leptin correlated with sICAM1 rho = 0.786, and sVCAM1 negatively with glycemia/insulin rho = −0.85. Significant differences were found between the groups regarding sVCAM1 (*p* = 0.0134), leptin (*p* = 0.0265) and all insulin resistance scores, with differences influenced by the subjects’ gender. In conclusion, although there are currently many unknown aspects of the release and the role of various adipokines, in particular chemerin, its implication in early glucose metabolism dysregulation disorders seems very likely.

## 1. Introduction

Diabetes and obesity currently represent public health issues [1,2,3], pandemic diseases [4], with a growing incidence [5,6,7,8], hundreds of millions of people being diagnosed with obesity or diabetes worldwide. More than 60% of the United States population are overweight or obese [9], and 463 million adults are diabetic across the world (according to 2019 IDF—International Diabetes Federation reports) [10]. A proportion of 85% of type 2 diabetic (T2DM) adults are also obese. The risk of diabetes is about nine times higher in obese subjects [11].

Early diagnosis and estimation of the risk of complications represent current objectives in order to reduce mortality and morbidity.

At this moment, obesity is described as a heterogeneous syndrome (determined by the genetic and environmental factors’ interaction) [8,12,13,14]. Previous studies have pointed out the different obesity types [15]: “metabolically healthy obesity”—MHO (defined by obesity presence without metabolic disturbance or metabolic syndrome features [8,12,16] and “metabolically unhealthy obesity—MUO” [8,12,16].

MHO patients, despite the fat mass excess, present a more favorable metabolic profile and lower, but still present, risk for late complications [16,17,18]. At the same time, the MHO concept is still controversial because of many discrepancies in relationship to its definition, prevalence (range between 2–50% in the previously reported data [16]), and etiopathogenesis, but, especially due to its tendency to eventually evolve into MUO [8,16].

Adipose tissue is a metabolically active [5,6], highly dynamic endocrine and immune organ [9,19,20,21,22,23] that secretes adipocytokines [24], which are involved in the regulation of lipid and carbohydrate metabolism, in the pathogenesis of insulin resistance and diabetes mellitus [4,5,14,23], in immunity, having at the same time a neuroendocrine function. Obesity is accompanied by an alteration of the adipokine profile (changes in the serum levels of adiponectin, leptin, resistin, chemerin, omentin, apelin, adipsin, vaspin, visfatin), as well as by the presence of inflammation (evidenced by changes in the adhesion molecules sICAM1—soluble Intercellular Adhesion Molecule 1 and sVCAM1—soluble Vascular Adhesion Molecule 1) [5,14].

Despite the large amount of data reported to date, there are still many unknown aspects related to the mode of action and the role of various molecules secreted by the adipose tissue, the interaction between them and their implication in the pathogenesis and the diagnosis of insulin resistance and endothelial dysfunction, obesity and diabetes mellitus [25]. The previously mentioned concepts (MHO, MUO) create more difficulties in understanding adiposity-related inflammation and neuroendocrine function, variant molecules’ expression being influenced by obesity phenotype.

The aim of this study was to evaluate the relationship between various adipocytokines, markers of inflammation, and insulin resistance (quantified by insulin resistance scores), as well as to monitor the way in which this relationship can be influenced by different combinations in the presence of obesity and diabetes mellitus.

## 2. Experimental Section

The current study was conducted in the Department of Cardiology of the Rehabilitation Hospital in Cluj-Napoca. Eighty-eight subjects with a mean age of 61.96 ± 10.15 years, including 66 (75%) women, were evaluated. All subjects underwent complete clinical examination, and the following was recorded: weight, height, body mass index (weight (kg)/(height * height [m^2^])), abdominal circumference (measured halfway between the last rib and the iliac crest), systolic and diastolic blood pressure (assessed after 15 min of rest, according to the recommendations of current guidelines). Cardiovascular risk factors (lipid fractions, obesity, smoking, diabetes, and hypertension) were assessed. The presence of diabetes mellitus was quantified based on the current criteria of the European Society of Cardiology [26]. Subjects were considered obese if body mass index (BMI) ≥ 30 kg/m^2^, hypertensive if they had blood pressure values ≥ 140/90 mmHg or if they were on hypotensive treatment, and dyslipidaemic if the serum total cholesterol ≥ 200 mg/dL or serum triglycerides ≥ 150 mg/dL (according to the 2019 European Society of Cardiology guidelines) [27]. Insulin resistance scores were calculated as follows: * homeostatic model assessment (HOMA index) = insulin (µU/mL) * glycemia (mg/dL)/405; * quantitative insulin sensitivity check index (QUICKI index = 1/[lg10 (insulin (µU/mL)) + lg10 (glycemia)); and * McAuley Score for measuring the Insulin Sensitivity Index = exp (3.29—0.25 * ln(Insulin)—0.22 * ln (BMI)—0.28 * ln (triglycerides)).

For each subject, the profile of serum adipokines (leptin, ghrelin, chemerin) was evaluated using the ELISA (enzyme-linked immunosorbent assay) method. The values of the adhesion molecules (soluble Intercellular Adhesion Molecule 1—sICAM1 and soluble Vascular Cell Adhesion Molecule 1—sVCAM1—in ng/mL) were measured using commercially available ELISA kits (R&D Systems Inc., Minneapolis, MN, USA).

Subjects with systemic or inflammatory diseases were excluded. The study protocol was approved by the local Ethics Committee (following the Declaration of Helsinki), and all subjects gave their written informed consent.

Statistical analysis of the data was performed using the statistical packages Medcalc version 10.3.0.0 (MedCalc Software, Ostend, Belgium) and SPSS for Windows (v16.0, IBM Corporation, Armonk, NY, USA). For quantitative variables, the normality of the distribution was evaluated using the Kolmogorov–Smirnov and D’Agostino–Pearson tests. The results were presented as the mean ± standard deviation, median values, respectively, for the quantitative variables (depending on the type of distribution), and as numbers and percentages for qualitative data. To assess the differences between variables, the independent sample *t*-test, Mann–Whitney test or *χ*^2^-test were used. The differences between groups were assessed using the ANOVA (analysis of variance) or Kruskal–Wallis test. Spearman and Pearson correlation coefficients were calculated. Optimum sensitivity, specificity, cut-off values, and area under receiver-operating characteristic (ROC) curves were assessed. A *p*-value < 0.05 was considered statistically significant.

## 3. Results

Eighty-eight subjects with a mean age of 61.96 ± 10.15 years, including 66 (75%) women, were evaluated. The mean age was 65.72 ± 10.04 years for men vs. 60.71 ± 9.94 years for women (*p* = 0.04). Of all subjects, 27.3% were diabetic (type 2—T2DM) and 35.3% were obese. The characteristics of the studied group are shown in Table 1.

Subjects were categorized in groups: group 1, obese + diabetic—13 subjects (14.8%); group 2, obese only—18 subjects (20.5%); group 3, diabetic only—11 subjects (12.5%); and group 4, non-diabetic non-obese—46 subjects (52.3%). The distribution of subjects in the four groups depending on gender is presented in Table 2, with no significant differences depending on subjects’ gender (*p* = NS).

Differences between groups (regarding adipokine levels, adhesion molecule levels, and insulin resistance scores) were assessed—data are presented in Table 3. Significant differences were found between groups regarding sVCAM1 (*p* = 0.0134), leptin (*p* = 0.0265), and all insulin resistance scores. The differences were influenced by subjects’ gender.

**Figure 1:**
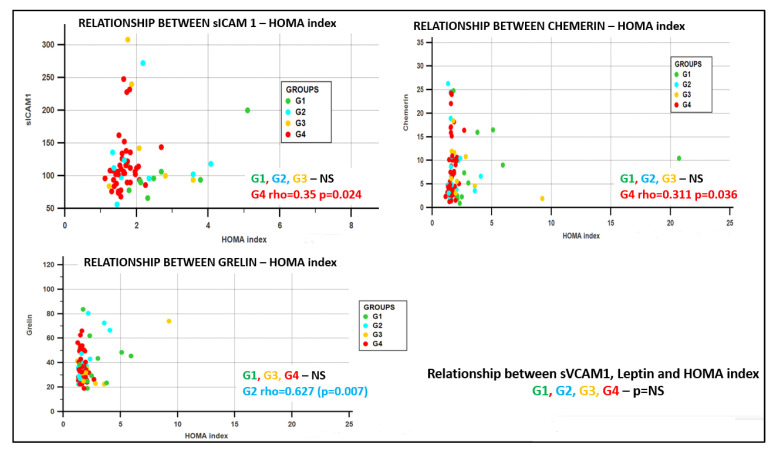
Relationship between the HOMA index (homeostatic model assessment) and adhesion molecules and adipokines. Panel a: relationship between sICAM1 (soluble Intercellular Adhesion Molecule 1) and HOMA index depending on the subjects’ groups; panel b: relationship between chemerin and HOMA index depending on the subjects’ groups; panel c: relationship between ghrelin and HOMA index depending on the subjects’ groups.

Significant correlations between adipokines, inflammation molecules, insulin resistance scores, anthropometric measurements, and biochemical parameters for the entire group as well as for the subgroups are presented in Table 4 and Figure 1.

We found significant correlations across all groups in the sICAM1–sVCAM1 rho = 0.426 (*p* < 0.001), and in the ghrelin–chemerin rho = −0.224 (*p* = 0.04).

We also found the following correlations in each group:

- Group G1 (obesity positive + diabetes positive), in the sICAM1–leptin rho = 0.786 (*p* = 0.03), sVCAM1–glycemia/insulin rho = −0.85 (*p* = 0.004);
- Group G2 (obesity positive + diabetes negative), in the sICAM1–sVCAM1 rho = 0.733 (*p* = 0.025), and in the sVCAM1-chemerin rho = 0.667 (*p* = 0.05);
- Group G3 (obesity negative + diabetes positive), no significant correlations;
- Group G4 (obesity negative + diabetes negative), in the sICAM1–HOMA rho = 0.35, *p* = 0.024, sICAM1–QUICKI rho = −0.35, *p* = 0.023, leptin–glycemia/insulin rho = −0.371 *p* = 0.024, ghrelin–chemerin rho =−0.350, *p* = 0.018, chemerin–HOMA rho = 0.311, *p* = 0.036, and in the chemerin–QUICKI rho = −0.31, *p* = 0.036.

Overall, the subjects with insulin resistance presented greater values of chemerin—9.05 ± 5.1 pg/mL (median value 8.2 pg/mL) vs. 8.6 ± 7.54 pg/mL (median value 5.6 pg/mL) in insulin-sensitive subjects. Even in a small subject sample, the aforementioned relationship was true in G1 (chemerin median value 9.7 pg/mL in insulin resistant vs. 2.5 pg/mL in insulin sensitive subjects) and in G4 (16.4 pg/mL vs. 7 pg/mL).

For chemerin, the greater determined area under the ROC curve was found in group G4 (obesity negative + diabetes negative). A chemerin value > 4.8 pg/mL was capable to identify the HOMA index > 2 subjects with a Se = 100%, Sp = 46.3%, area under the ROC (AUROC) = 0.688 (in women, AUROC = 0.719; however, we were not able to perform the test with the men, due to the small sample size of male subjects). The prediction capacity was better in G4 (AUROC = 0.688, Sp = 46.3%) vs. the entire sample (AUROC = 0.508, Sp = 19.05%).

## 4. Discussion

The adoption of the Western European diet and other similar lifestyle changes have caused an unfortunate increase in cases of diabetes and obesity. This change has had significant consequences for the overall health of the population and increased the financial burden on our health systems. In many cases, the diagnosis of T2DM is late, and patients have already developed complications from the illness [28].

As is currently well known, adipose tissue itself represents an endocrine organ [29,30,31,32]. Recent studies have shown that obesity and the increase in the size and number of adipocytes are associated with an alteration of lipid and carbohydrate metabolism [31,33,34], insulin resistance and systemic inflammation [33]. Adipose tissue causes these changes through the secretion of biologically active molecules: over 600 adipokines (such as leptin, resistin, apelin, vaspin, omentin, visfatin, adiponectin, chemerin, etc.), cytokines, both pro-inflammatory and anti-inflammatory, interleukins- IL (IL1, IL6), chemokines (IL8, MCP 1- monocye chemoattractant protein-1), vasoactive substances, interferon, and hormone-like action proteins [29,30,31,32,33,34,35]. The increase in the level of proatherogenic substances, with a pro-inflammatory effect, might trigger a cascade of insulin resistance [34], as well as the relationship between obesity and diabetes and vascular complications, respectively [34]. However, to date there are no data to explain the exact chronology of this process [34].

In the development of atherosclerotic vascular damage induced by obesity or diabetes, endothelial dysfunction occurs at an early, subclinical stage [36] by activating inflammation and initiating the adhesion cell expression [37]. There are still many controversies surrounding the methods of quantifying early endothelial dysfunction (including a genetic evaluation to identify potential risk) [38]. The assessment of the role of adhesion molecules is questioned [37]. Previous studies have shown that the family of adhesion molecules is extremely heterogeneous. While sICAM1 is expressed in endothelial cells (with low levels detected even when there are no alterations of these) [39], smooth muscle cells, and epithelial cells, sVCAM1 is an indicator of plaque activity (being detected only in endothelial cells) [37,39].

An increase in the levels of sICAM1 and sVCAM1 was evidenced in patients with insulin resistance [40], and in those who will later develop [41,42] or already present diabetes [40,43,44]. sICAM1 values are generally higher in women with insulin resistance vs. men [39]. In this study, of the two adhesion molecules, sICAM1 was correlated with insulin resistance indices, but only in G4 (O-D-). No significant differences in the sICAM1 values between groups were found. Regarding sVCAM1, diabetic subjects with obesity had significantly higher values compared to normal weight subjects (G1 vs. G3, *p* < 0.05). Both overall and in the case of analysis by groups, significant correlations were detected between the values of two adhesion molecules, similarly to previously reported data [39,45].

Leptin (the first adipocyte hormone identified), is a 167 amino acid hormone secreted by adipose tissue. It has an influence on dietary intake [14,24], body weight, and adipose deposits [30] through a direct effect on the hypothalamus [34], which induces the sensation of satiety [35]. Leptin has pro-inflammatory activity (activation of macrophages, T and NK cells, the release of cytokines, interleukins IL6 and IL12 [30,35]), while simultaneously playing a role in endothelial cell proliferation and migration [46], platelet aggregation [46], and promotion of endothelial dysfunction (stimulation of the release of adhesion molecules, M-CSF (macrophage colony-stimulating factor), promotion of cholesterol accumulation, angiogenesis [30] and atherogenesis). Obese patients have increased serum levels (due to increased secretion from the adipose tissue), proportional to insulin levels [30], while leptin resistance develops over time [30,34]. According to the literature [47], higher leptin levels were found in women than in men, and the presence of obesity (associated or not with the presence of diabetes mellitus) determined significantly higher values of serum leptin in both genders. In group G1, we detected a correlation between the levels of leptin and adhesion molecules, which evidenced significant inflammation present in these conditions.

Ghrelin, a stomach-derived hormone (produced by X/A-like cells within the gastric oxyntic glands of the stomach) [3], is a key player in the regulation of appetite and energy homeostasis [48]. It is also involved in glucose metabolism and homeostasis [48], insulin sensitivity, in the development of diabetes [49,50], and in adipogenesis [49]. Ghrelin is considered to play a bimodal role, initially proatherogenic (in the early formation of plaques), and later antiatherogenic (as the atherosclerotic disease progresses) [49]. It may also have pro-inflammatory and anti-inflammatory properties [49] and protective effects on the heart [3].

Ghrelin secretion shows an alteration of the physiological pattern in patients with insulin resistance or obesity [48]. Low ghrelin levels have been recorded in diabetic patients [49] as well as in obese patients [48,51,52,53,54]. The literature advances the idea that secretion would be inhibited by hyperinsulinemia and hyperleptinemia [52]. Low ghrelin levels are associated with unfavorable prognosis and increased global cardiovascular risk [52]. This study found no significant differences between the four groups (probably due to the bimodal role); surprisingly, obese non-diabetic subjects had high serum ghrelin levels. In group G2, ghrelin was significantly correlated with insulin resistance.

Chemerin, a recently discovered adipokine [55] (also known as tazarotene-induced gene 2 (TIG2) and retinoic acid receptor responder 2 (RARRES2) [31]) is a 163 amino acid pre-proprotein that is secreted as a 143 amino acid (18 kDa) proprotein and subsequently undergoes C-terminal cleavage [32,33]. It has recently been discovered that chemerin has at least three receptors: G protein-coupled receptor CMKLR1 (chemokine-like receptor 1 or ChemR23), G protein-coupled receptor 1 (GPR1) and (C-C motif) receptor-like (CCRL) 2 [56,57,58]. CMKLR1 is expressed mainly in adipose tissue (higher levels in white compared to brown adipose tissue), bone, lung, brain, heart, and placenta [55,59]. Chemerin binds to orphan CMKLR1 [24,60], activates the three Gαi subtypes, and the two Gαo isoform [56], exercising in this way main biological functions [57,60]. CMKLR1 activation is responsible for intracellular calcium release and a reduction in cAMP (cyclic adenosine monophosphate) accumulation [59]. On the other hand, a loss of CMKLR1 has been reported to be associated with reduced body mass and adiposity [59,61], and its upregulation is found in obese patients [56].

Chemerin plays a role in adipogenesis [6,8,29,55,56,62], preadipocyte differentiation, adipocyte development and metabolism [29,31,57,63,64,65], insulin sensitivity [6,56], and in lipid and glucose metabolism [29,55,58,66,67]. It is a pro-inflammatory agent [8,16,33,55,65], a chemoattractant for various types of cells [8,16,33,64,65], and modulates immune system function [31,56].

Initially, it was believed that the secretion of chemerin increases significantly in parallel to adipogenesis. It was assumed that the alteration of this process, or the change in the expression of the chemerin receptor CMKLR1 or chemerin–CMKLR1 signaling, affected adipocyte differentiation [7,56,68,69], impaired glucose homeostasis [70] and glucose-stimulated pancreatic insulin release [7], affected insulin sensitivity [71], and even modified the genes involved in lipid and glucose metabolism [7]. Recently, given chemerin’s multiple roles, a new hypothesis was formed—chemerin itself can be a link between inflammation, obesity and atherosclerosis [72].

The data published to date reflect the fact that serum chemerin values are correlated with body mass index, serum triglyceride levels and inversely with HDL cholesterol values [29,31,33,55,62,67,73,74,75]. In addition, it was reported that elderly people had higher chemerin values [31,73,74], but without significant differences depending on the patients’ gender [31,76]. This study found no significant differences depending on the subjects’ sex either.

The relationship between the chemerin and insulin resistance/insulin sensitivity is not completely understood; some studies report a direct association with insulin resistance [62,67,74,77,78,79,80,81,82], while others refute it, saying that chemerin is actually involved in the regulation of insulin sensitivity [7,55,65,83,84]. At the same time, the information reported to date regarding the relationship between chemerin values and the presence of obesity or diabetes is contradictory. There are data referring to the increase in serum chemerin values in obese patients [6,24,29,55,56,58,62,64,72,75,85], in obese non-diabetic patients [75,86], in diabetic patients [24,29,55,58,64,74,80,83,85,87], in obese diabetic patients [81], and in patients with metabolic syndrome [31,78]. On the other hand, some studies highlight the absence of changes in chemerin values [88] or even their decrease in diabetic patients [63], or the absence of significant differences between obese and non-obese diabetic patients [74].

The reasons for these discrepancies are under evaluation. The expression of chemerin might be related to a threshold between adipogenesis and inflammation. During early adipogenesis, chemerin promotes insulin sensitivity, but increased adipogenesis during obesity makes chemerin release proinflammatory cytokines.

In our study, in the group of non-diabetic non-obese subjects, there was a positive correlation between chemerin and the HOMA index. This finding supports the previously mentioned idea that chemerin might be an early marker of insulin resistance, as it is capable of predicting the development of metabolic syndrome [16] or diabetes [7,79,87], detecting the alteration of insulin sensitivity [49,50,66], and discriminating subjects with subclinical diabetes [66,89]. The best predictive capacity for insulin resistance was also found in the non-diabetic non-obese group.

In time, increased inflammation leads to insulin resistance and subsequent diabetes. Even if the clinical onset of diabetes is preceded by the development of insulin resistance, this is extremely difficult to quantify, so identifying early markers might be a key step in the management of diabetes.

Moreover, it must be considered that not all obese patients have insulin resistance (about 25% of obese subjects are insulin sensitive, being classified, as we mentioned, into metabolically healthy obese) [9,82,90], and remain insulin-sensitive for a period [9,91]. These MHO patients seem to present lower chemerin levels vs. MUO patients [16]. This is in accordance with our findings that registered chemerin values are lower in obese-diabetic patients with lower values of the HOMA index.

It should be mentioned that serum chemerin values can also be influenced by the medication that a patient is taking. Some published studies [79,92] reported the fact that pioglitazone and metformin (most likely due to increased insulin sensitivity) [93] caused a decrease in serum chemerin values in patients with T2DM. In the studied group, patients with T2DM did not show higher chemerin values, which might be attributed to the aforementioned influence of medication (unfortunately, relevant information about the medication administered to the investigated subjects was not collected).

The interactions between different adipokines create a complex picture, with various interrelations and the effects of adipokines being both synergistic and antagonistic. Previous studies evidence a correlation between chemerin and leptin levels [33,74], as well as between chemerin and markers of systemic inflammation (C-reactive protein, interleukin 6, and TNF alpha) [33,66,67,74,94]. We did not detect a correlation between chemerin and leptin, but depending on the studied subgroup, chemerin was correlated with sVCAM1. To the best of our knowledge, there are no reported data regarding the relationship between chemerin and ghrelin. We found that the study subjects as a whole showed a significant inverse correlation between chemerin and ghrelin, which was maintained in G4 (subclinical changes and early insulin resistance caused an early increase in chemerin and a decrease in ghrelin).

By synthesizing the presented data, we can advance the hypothesis, supported by other authors [33,57,87], that chemerin is a possible link between obesity–inflammation–diabetes–atherosclerosis, and that its release could be much earlier than that of other adipokines.

Limitations of the study include the small number of subjects. We also have to mention the discrepancy between the number of women and the number of men. Our subjects were consecutive hospital patients who met our criteria, so our control over the diversity of subjects was limited. Further studies are necessary to quantify the influence of the subjects’ gender on adipokines–obesity–diabetes interaction. To at least partially support our results (in both genders), we want to mention the fact that no significant differences were found between the genders regarding metabolic syndrome features (including insulin resistance/sensitivity scores), with the only exception of the lipid profile (HDL cholesterol). Furthermore, due to the small number of subjects, we were unable to investigate how obesity or gender might influence chemerin behavior. Our data did not allow us to study how a patient’s medication might influence insulin resistance, either.

The current study opens new research directions to identify changes in “active molecules” as early as possible, which would not only allow early diagnosis, but especially the prevention of certain diseases.

In conclusion, there are still many questions surrounding the release and the role of various adipokines, in particular chemerin. During early adipogenesis, it is possible for chemerin to promote insulin sensitivity and to provide useful information regarding the early diagnostic of glucose metabolism dysregulation disorders.

## Figures and Tables

**Table 1:** Subjects’ characteristics.

		Global	Women	Men	*p*
Number		88	66	22	
Age (years)		61.96 ± 10.15	60.71 ± 9.94	65.72 ± 10.04	0.04
Body mass index (kg/m^2^)		28.85 ± 4.22	28.88 ± 4.39	28.73 ±3.76	NS
Obesity No (%)	Yes	31 (35.3)	24 (36.36)	7 (31.81)	NS
	No	57 (64.7)	42 (63.63)	15 (68.18)	
Abdominal circumference (cm)		98.04 ± 10.41	96.27 ± 10.40	103.27 ± 8.70	0.003
Systolic blood pressure (mmHg)		131.59 ± 16.28 (130)	131.43 ± 16.47 (130)	132.04 ± 16.08 (130)	NS
Diastolic blood pressure (mmHg)		82.78 ± 15.38 (80)	83.56 ± 16.88 (80)	80.45 ± 9.5 (80)	NS
Hypertension No (%)	Yes	70 (79.5)	53 (80.30)	17 (77.27)	NS
	No	18 (20.5)	13 (19.7)	5 (22.73)	
Current smokers No(%)	Yes	15 (17)	10 (15.15)	5 (22.72)	NS
	No	73 (83)	56 (84.85)	17 (77.28)	
Diabetes No (%)	Yes	24 (27.3)	16 (24.24)	8 (36.36)	NS
	No	64 (72.7)	50 (75.75)	14 (63.63)	
Glycemia (mg/dL)		100.35 ± 34.77	101.34 ± 38.87	97.36 ± 17.83	NS
Dyslipidemia	Yes	59 (67)	48 (72.72)	11 (50)	0.08
	No	29 (33)	18 (27.27)	11 (50)	
Total cholesterol (mg/dL)		213.07 ± 52.82	222.03 ± 48.09	186.22 ± 58.24	0.014
LDL cholesterol (mg/dL)		137.05 ± 41.34	142.87 ± 37.60	119.59 ± 47.72	0.045
Triglycerides (mg/dL)		154.50 ± 69.15	158.65 ± 73.22	142.04 ± 54.81	NS
HDL cholesterol (mg/dL)		42.82 ± 9.21	44.34 ± 8.80	38.27 ± 9.10	0.006
sICAM1 (ng/mL) *		120.36 ± 51.11 (106.00)	123.58 ± 46.74 (108.00)	111.77 ± 61.97 (95.00)	0.034
sVCAM1 (ng/mL) *		1106.48 ± 452.89 (998.00)	1114.33 ± 460.16 (998.00)	1085.55 ± 445.17 (988.00)	NS
Leptin (pg/mL) *		26,201.16 ± 23,946.59 (20,195.00)	32,758.40 ± 24,533.35 (24,155.00)	8168.75 ± 7559.82 (5150.00)	<0.0001
Ghrelin (pg/mL) *		39.16 ± 17.34 (34.50)	40.37 ± 19 (35)	35.70 ± 10.97 (33.75)	NS
Chemerin (pg/mL) *		8.60 ± 7.22 (6.30)	8.97 ± 7.72 (6.3)	7.47 ± 5.44 (5.95)	NS
Insulin (µU/mL)		8.50 ± 5.19 (7.40)	8.88 ± 5.96 (7.4)	7.38 ± 0.5 (7.25)	0.049
HOMA index *		2.23 ± 2.29 (1.67)	2.38 ± 2.62 (1.66)	1.78 ± 0.39 (1.78)	NS
QUICKI index *		0.34 ± 0.01 (0.35)	0.34 ± 0.02 (0.35)	0.35 ± 0.01 (0.34)	NS
McAuley Score		1.93 ± 0.28	1.90 ± 0.29	2.00 ± 0.24	NS
Glycemia/insulin *		12.55 ± 3.62 (12.16)	12.35 ± 3.99 (11.83)	13.17 ± 2.13 (12.32)	0.05

LDL cholesterol = low-density lipoprotein; HDL cholesterol = high-density lipoprotein cholesterol; sICAM1 = soluble Intercellular Adhesion Molecule 1; sVCAM1 = soluble Vascular Adhesion Molecule 1; HOMA index = homeostatic model assessment; Quicki index = quantitative insulin sensitivity check index; * does not respect the normality distribution; data are presented as the mean ± standard deviation (median value); for categorical data: number (percentage), *p* was calculated with Student’s test, Mann–Whitney test, or χ2 test; NS (not statistically significant) *p* >0.05.

**Table 2:** Distribution of subjects depending on the presence of diabetes and obesity.

	Group 1	Group 2	Group 3	Group 4
	O + D +	O + D -	O - D +	O -D -
Number of subjects	13	18	11	46
Women	10 (76.9)	14 (77.77)	6 (54.54)	36 (78.6)
Men	3 (23.07)	4 (22.22)	5 (45.450	10 (21.7)

Data are presented as numbers (percentages).

**Table 3:** Differences between the groups regarding the different parameters (adipokines, adhesion molecules, insulin resistance scores).

GROUPS	Group 1 O + D + 13 Subjects	Group 2 O + D - 18 Subjects	Group 3 O - D + 11 Subjects	Group 4 O - D - 46 Subjects	Global *p* Trend (Sig Diff between Groups)	*p* Trend Women (Sig Diff between Groups)	*p* Trend Men (Sig Diff Groups)
Age	62.23 ± 10.05	53.83 ± 7.95	69.18 ± 10.6	63.34 ± 9.06	<0.001 G1 vs. G2, G2 vs. G3, G2 vs. G4	<0.001 G1 vs. G2, G2 vs. G3, G2 vs. G4, G3 vs. G4	NS
sICAM1 (ng/mL) *	104.22 ± 38.70 (94)	123.77 ± 59.99 (112)	167.42 ± 85.54 (142)	115.12 ± 40.63 (106)	NS	NS	NS
sVCAM1 (ng/mL) *	790.88 ± 164.87 (742)	995.55 ± 297 (928)	1562.57 ± 858 (1140)	1122.24 ± 371 (1054)	0.0134 G1 vs. G3, G1 vs. G4	0.0385 G1 vs. G3, G1 vs. G4	NS
Leptin (pg/mL) *	38,121.25 ± 24,875 (32,810)	40,318.88 ± 28,541 (36,650)	33,893.33 ± 39,946 (14,505)	18,942.43 ± 16,474 (15,950)	0.0265 G1 vs. G4, G2 vs. G4	0.0048 G1 vs. G4, G2 vs. G4 G3 vs. G4	0.0175 G1 vs. G4, G2 vs. G3, G2 vs. G4
Ghrelin (pg/mL) *	38.30 ± 18.38 (29.5)	43.61 ± 15.90 (39)	34.80 ± 14.93 (31.7)	38.70 ± 18.22 (34.5)	NS	NS	NS
Chemerin * (pg/mL)	7.98 ± 7.22 (5.2)	8.42 ± 7.55 (5.8)	7.27 ± 5.24 (5.6)	9.15 ± 7.64 (7.15)	NS	NS	0.0259 G1 vs. G3, G1 vs. G4, G2 vs. G4
Insulin	12.51 ± 12.65 (7.6)	8.07 ± 2.19 (7.4)	8.17 ± 1.66 (7.4)	7.60 ± 0.92 (7.3)	0.023 G1 vs. G2, G1 vs. G3, G1 vs. G4	0.023 G1 vs. G2, G1 vs. G4	NS
HOMA index *	4.24 ± 5.12 (2.48)	1.90 ± 0.78 (1.59)	2.69 ± 2.26 (1.87)	1.66 ± 0.28 (1.64)	0.0002 G1 vs. G2, G1 vs. G4, G3 vs. G4	0.0002 G1 vs. G2, G1 vs. G4, G2 vs. G3, G3 vs. G4	NS
QUICKI index *	0.32 ± 0.02 (0.33)	0.35 ± 0.01 (0.35)	0.33 ± 0.02 (0.34)	0.35 ± 0.008 (0.35)	0.0002 G1 vs. G2, G1 vs. G4, G3 vs. G4	0.0001 G1 vs. G2, G1 vs. G4, G2 vs. G3, G3 vs. G4	NS
McAuley Score	1.73 ± 0.38	1.90 ± 0.27	1.88 ± 0.27	2.01 ± 0.22	0.014 G1 vs. G4	0.006 G1 vs. G4	NS
Glycemia/insulin *	13.86 ± 5.34 (14.42)	12.19 ± 4.10 (11.60)	14.79 ± 5.5 (13.75)	11.79 ± 1.54 (11.76)	0.0076 G1 vs. G2, G1 vs. G4, G2 vs. G3, G3 vs. G4	0.06	NS

sICAM1 = soluble Intercellular Adhesion Molecule 1; sVCAM1 = soluble Vascular Adhesion Molecule 1; HOMA index = homeostatic model assessment; Quicki index = quantitative insulin sensitivity check index; * does not respect the normality distribution; for *p* trend; ANOVA (analysis of variance) or Kruskal–Wallis test was used; NS (not statistically significant) *p* >0.05, G1 = group 1, G2 = group 2, G3 = group 3, G4 = group 4.

**Table 4:** Correlations between the parameters (at the global level and at a specific group level).

		sICAM1	sVCAM1	LEPTIN	GHRELIN	CHEMERIN	HOMA	QUICKI	McAuley Score	GLYC/INS
**BMI**	Global	0.045	−0.31 *	0.402 **	0.10	−0.28	0.338 **	−0.339 **	−0.328 **	0.11
	G1	−0.183	0.083	−0.19	−0.209	−0.247	−0.005	0.005	−0.204	−0.27
	G2	−0.3	−0.4	0.3	0.04	−0.15	0.01	−0.01	0.179	0.14
	G3	0.75 *	0.107	0.02	−0.17	0.39	0.18	−0.18	0.139	0.39
	G4	0.19	−0.09	0.179	0.05	0.142	0.319 *	−0.33 *	−0.365 *	−0.125
**WC**	Global	0.034	−0.22	0.098	0.086	−0.36	0.334 **	−0.334 **	−0.225 *	0.220 *
	G1	−0.08	−0.21	−0.31	0.166	−0.43	−0.088	0.088	0.07	0.441
	G2	0.025	0.084	0.377	0.006	−0.09	0.005	−0.005	−0.05	0.18
	G3	0.306	−0.306	−0.493	−0.134	0.11	0.128	−0.128	0.398	0.604
	G4	0.142	−0.03	−0.18	0.069	0.224	0.229	−0.242	−0.191	0.08
**TC**	Global	0.092	−0.187	0.249	0.04	0.087	−0.009	0.012	−0.495 **	−0.14
	G1	0.75 *	−0.133	0.23	0.236	0.165	0.40	−0.401	−0.628 *	0.148
	G2	0.517	−0.033	0.667 *	0.061	0.32	0.04	−0.04	−0.653 **	−0.512 *
	G3	−0.28	−0.286	0.60	−0.05	−0.345	−0.05	0.05	−0.37	0.009
	G4	−0.062	−0.147	0.124	−0.037	−0.038	−0.106	0.112	−0.514**	−0.08
**LDL-C**	Global	0.133	−0.145	−0.140	0.004	0.042	0.05	−0.49	−0.384 **	−0.103
	G1	0.603	−0.293	0.036	0.124	0.047	0.316	−0.316	−0.536	0.206
	G2	0.750 *	0.383	0.317	0.126	0.198	0.190	−0.19	−0.629 **	−0.583 *
	G3	−0.179	−0.429	0.771	−0.03	−0.255	−0.023	0.023	−0.17	0.182
	G4	−0.001	−0.162	0.11	−0.024	−0.06	−0.017	0.022	−0.409 **	−0.05
**HDL-C**	Global	0.176	0.054	0.169	0.178	0.196	−0.254 *	0.256 *	0.096	−0.205
	G1	0.795 *	0.059	0.503	0.515	0.454	0.263	−0.263	−0.338	0.069
	G2	0.343	0.393	0.803 **	−0.224	0.562 *	−0.221	0.221	−0.08	0.09
	G3	−0.286	0.01	0.314	0.330	−0.40	−0.2	0.20	−0.08	0.05
	G4	0.075	0.009	0.081	0.118	0.086	−0.166	0.167	0.325 *	−0.219
**TG**	Global	−0.161	−0.22	0.08	0.052	0.052	0.145	−0.143	−0.863 **	−0.06
	G1	0.45	0.1	0.286	0.264	0.407	0.385	−0.385	−0.791 **	−0.038
	G2	0.183	−0.283	0.233	0.432	0.22	0.125	−0.125	−0.91 **	−0.677 **
	G3	−0.714	−0.25	0.371	0.212	−0.582 *	0.291	−0.291	−0.957 **	−0.236
	G4	−0.295	−0.129	−0.08	−0.175	0.021	−0.017	0.026	−0.901 **	0.113

sICAM1 = soluble Intercellular Adhesion Molecule 1; sVCAM1 = soluble Vascular Adhesion Molecule 1; HOMA index = homeostatic model assessment; Quicki index = quantitative insulin sensitivity check index; **GLYC/INS = ratio between glycemia and insulin;** Spearman coefficient or Pearson coefficient was calculated (depending on distribution type—normal or not); * correlation was significant at the 0.05 level (2-tailed). ** correlation was significant at the 0.01 level (2 tailed). TC = total cholesterol, LDL-C = LDL cholesterol, HDL-C = HDL cholesterol, TG = triglycerides, BMI = body mass index, WC = waist circumference, G1 = group 1, G2 = group 2, G3 = group 3, G4 = group 4.
